# Poly (Octadecyl Methacrylate-Co-Trimethylolpropane Trimethacrylate) Monolithic Column for Hydrophobic in-Tube Solid-Phase Microextraction of Chlorophenoxy Acid Herbicides

**DOI:** 10.3390/molecules24091678

**Published:** 2019-04-29

**Authors:** Wenbang Li, Fangling Wu, Yongwei Dai, Jing Zhang, Bichen Ni, Jiabin Wang

**Affiliations:** 1 Institute of Biomedical and Pharmaceutical Technology, Fuzhou University, Fuzhou 350002, China; N165720012@fzu.edu.cn (W.L.), flwu16@fudan.edu.cn (F.W.); N185720006@fzu.edu.cn (B.N.); 2 Fuzhou Jiachen Biotechnology Co., Ltd., Fuzhou 350008, China; daiyoungwei@163.com; 3 Fujian Inspection and Testing Centre for Agricultural Product Quality and Safety, Fuzhou 350003, China; fjsy0591@sina.com

**Keywords:** chlorophenoxy acid herbicides, HPLC, hydrophobic in-tube solid-phase microextraction, poly (OMA-co-TRIM) monolithic column, rice grains

## Abstract

Chlorophenoxy acid herbicides (CAHs), which are widely used on cereal crops, have become an important pollution source in grains. In this work, a highly hydrophobic poly (octadecyl methacrylate-co-trimethylolpropane trimethacrylate) [poly (OMA-co-TRIM)] monolithic column has been specially prepared for hydrophobic in-tube solid-phase microextraction (SPME) of CAHs in rice grains. Due to the hydrophobicity of CAHs in acid conditions, trace CAHs could be efficiently extracted by the prepared monolith with strong hydrophobic interaction. Several factors for online hydrophobic in-tube SPME, including the length of the monolithic column, ACN and trifluoroacetic acid percentage in the sampling solution, elution volume, and elution flow rate, were investigated with respect to the extraction efficiencies of CAHs. Under the optimized conditions, the limits of detection of the four CAHs fell in the range of 0.9–2.1 μg/kg. The calibration curves provided a wide linear range of 5–600 μg/kg and showed good linearity. The recoveries of this method ranged from 87.3% to 111.6%, with relative standard deviations less than 7.3%. Using this novel, highly hydrophobic poly (OMA-co-TRIM) monolith as sorbent, a simple and sensitive online in-tube SPME-HPLC method was proposed for analysis of CAHs residue in practical samples of rice grains.

## 1. Introduction

In order to fulfill the world’s growing demand for food, herbicide application to cereal crops is imperative. Because of their low cost, high weeding effectiveness and little effect on cereal crops, chlorophenoxy acid herbicides (CAHs), represented by 2,4-dichlorophenoxyacetic acid (2,4-D), are extensively used on cereal crops for the selective control of most broadleaf weeds [1]. For the high absorption of CAHs by crops, CAHs have become an important pollution source in cereal grains, which cause potential pollution to food with further potential toxicity against humans. In June 2015, the World Health Organization's International Agency for Research on Cancer confirmed its 1987 classification of 2,4-D as a possible carcinogen [2]. Men who work with 2,4-D are at risk for abnormally shaped sperm and thus fertility problems [3]. Therefore, it has become important work to detect and monitor the residual level of CAHs in cereal grains by developing reliable analytical methods that are simple, highly sensitive, and cost-effective.

To date, the main techniques for the quantitative detection of CAHs in different samples include gas chromatography (GC) [4,5], high performance liquid chromatography (HPLC) [6,7,8], surface-enhanced raman spectroscopy (SERS) [9], capillary electrophoresis (CE) [10,11], and enzyme-linked immunosorbent assay (ELISA) [12]. Among them, GC and HPLC are generally adopted as the determination methods in many standards [13,14]. When detected by GC, the additional derivatization of CAHs is necessary. Thus, HPLC is more simple and suitable for the determination of CAHs. On the other hand, for the low concentrations and matrix interferences, sample preparation is indispensable prior to the chromatographic analysis. Several sample preparation methods, including liquid–liquid extraction [15], soxhlet extraction [16], solid-phase extraction (SPE) [17,18], suspended liquid-phase microextraction [19], molecularly imprinted solid-phase extraction [20,21], dispersive micro-solid phase extraction [22], matrix solid-phase dispersion [23], and microwave-assisted solvent extraction [24,25], have been carried out for the extraction of CAHs from a variety of matrixes. However, considerable amounts of time and solvent are required in these sample preparation processes.

Solid-phase microextraction (SPME), due to its advantages of high sensitivity, solvent-free extraction, simplicity, and easy online coupling to HPLC, has been recognized as a reliable method of sample preparation, especially for analysis in complex sample matrixes (e.g., biological and food samples) [26]. As the core of SPE or SPME, the selection of the appropriate sorbent is crucial. Diverse SPE sorbents, such as highly cross-linked polystyrene-divinylbenzene sorbent [17], molecularly imprinted polymers [20,21], and cationic gemini surfactant-resorcinol-aldehyde resin [22], have been utilized for the extraction of CAHs. Monolithic columns, which could be synthesized in situ in the capillaries or tubes, have attracted increasing interests as an SPME sorbent because of their inherent advantages of facile preparation, diverse surface chemistry, increased enrichment capacity, and high porosity [27]. Recently, a multiple monolithic fiber which was reinforced by graphene has been proposed for extraction of CAHs [28]. In our previous work, a poly (octadecyl methacrylate-co-ethylene dimethacrylate) [poly (OMA-co-EDMA)] monolithic column was fabricated for the extraction of CAHs from water samples [29]. Nevertheless, due to the higher complexity of sample matrixes of cereal grains than water, the unsatisfied extraction efficiency and worse renewability of poly (OMA-co-EDMA) monolith occurred and further showed its inapplicability facing the complex samples of cereal grains. Considering the hydrophobicity of CAHs in acid conditions, the monolithic column with higher hydrophobicity should be favored to enhance extraction efficiency for SPME. 

To this end, trimethylolpropane trimethacrylate (TRIM) with higher hydrophobicity was specially selected as an alternative crosslinker to fabricate a novel hydrophobic poly (octadecyl methacrylate-co-trimethylolpropane trimethacrylate) [poly (OMA-co-TRIM)] monolithic column for an online hydrophobic in-tube SPME of CAHs in rice grains. The characterization of the poly (OMA-co-TRIM) monolithic column was carried out in detail. Important SPME parameters, including the length of the monolithic column, the percentage of trifluoroacetic acid and acetonitrile (ACN) in sampling solution, elution volume, and elution flow rate, were optimized to obtain the best extraction efficiency. Under the optimized condition, an online in-tube SPME-HPLC method using the poly (OMA-co-TRIM) monolithic column as the sorbent was developed and applied for the determination of trace CAHs residue in rice grains.

## 2. Results and Discussion

### 2.1. Characterization of the Monolithic Column for Hydrophobic in-Tube SPME

#### 2.1.1. Morphology

**Figure 1:**
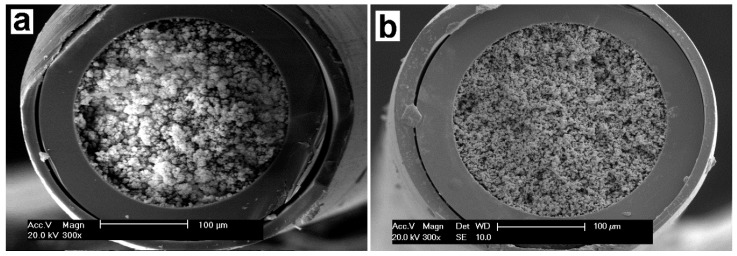
SEM images of (**a**) the poly (OMA-co-TRIM) monolithic column and (**b**) the poly (OMA-co-EDMA) monolithic column.

In this work, the detailed morphology of the poly (OMA-co-TRIM) monolithic column (Figure 1a) was captured by scanning electron microscope (SEM). In this SEM image, there were many mesopores and flow-through channels inlaid in the network skeleton of column bed. When compared with poly (OMA-co-EDMA) monolith in Figure 1b, the flow-through channels in the poly (OMA-co-TRIM) monolith were larger; they could provide large flow paths for sampling solutions and elution solutions to pass through in a fast flow rate, which would be favorable for the further extraction process.

#### 2.1.2. Permeability and Porosity

The permeability of the obtained monolith was investigated. A 5 cm column cut from the prepared monolithic column was connected to a μHPLC pump and the backpressure was measured by pumping water or ACN. The permeability (K) of the column was calculated with Darcy’s equation, K = FηL/(πr^2^∆P) [30], where F is the flow rate of mobile phase, η is the viscosity of mobile phase, L is the effective length of column, r is the inner radius of column, and ∆P is the pressure drop of column. The measured permeability of the poly (OMA-co-TRIM) monoliths were 1.25 × 10^−13^ m^2^ by using ACN as the mobile phase and 2.03 × 10^−13^ m^2^ by using water as the mobile phase. When measured in the same conditions, the permeability of the poly (OMA-co-EDMA) monoliths were 1.90 × 10^−13^ m^2^ and 2.80 × 10^−13^ m^2^, respectively. This result indicated that the permeability of this prepared poly (OMA-co-TRIM) monolith was better than the reported poly (OMA-co-EDMA) monolith and further showed its feasibility for in-tube SPME.

In addition, the pore size distribution of the poly (OMA-co-TRIM) monolithic column was also characterized by the mercury intrusion method (Figure S1). The most probable pore size of the monolith was 0.92 μm. Furthermore, the specific pore volume of the monolith was 1.36 mL/g, and the porosity was 56.34%. These data indicated the high porosity of the obtained poly (OMA-co-TRIM) monolith. 

#### 2.1.3. Loading Capacity

**Figure 2:**
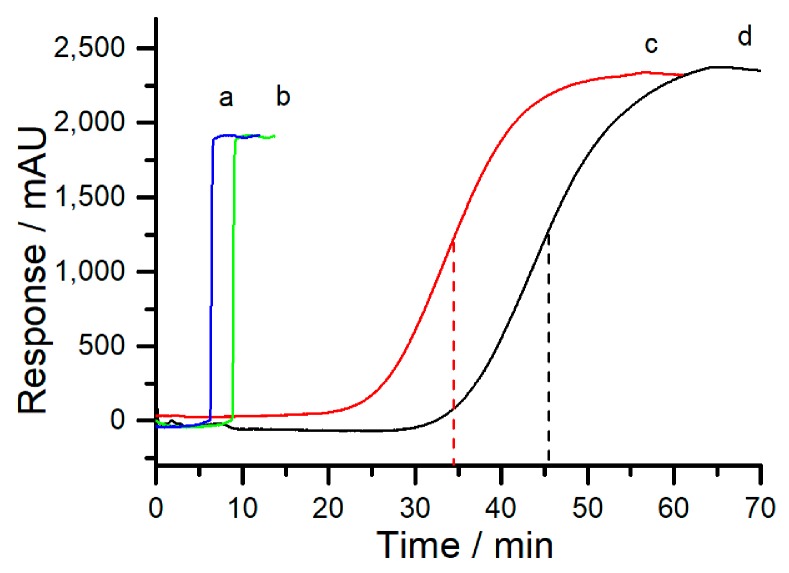
Breakthrough curves on different monoliths: (a) For thiourea (marker for void time) on poly (OMA-co-TRIM) monolith; (b) for thiourea (marker for void time) on poly (OMA-co-EDMA) monolith; (c) for 2,4-D on the poly (OMA-co-EDMA) monolith; (d) for 2,4-D on the poly (OMA-co-TRIM) monolith. Experimental conditions: The monolith, 20 cm × 250 μm i.d.; mobile phase, 98% (*v*/*v*) 0.1% trifluoroacetic acid (TFA) solution and 2% (*v*/*v*) ACN; pump flow rate, 10 μL/min; the concentraction of thiourea, 200 ng/mL; the concentraction of 2,4-D, 200 ng/mL; UV detection wave, 223 nm.

In this work, breakthrough curves for 2,4-D using frontal analysis were utilized to evaluate the loading capacity of the obtained monolith. As shown in Figure 2, the breakthrough curves of 2,4-D by using poly (OMA-co-TRIM) monolith and poly (OMA-co-EDMA) monolith both exhibited an obvious rise, demonstrating the typical kinetic adsorption. The void time for calculation was examined by flushing 200 ng/mL thiourea in the same monolithic column and subtracting from the total consumed time for saturating the monolithic column. The saturation times for 2,4-D on the above two monolithic columns were 24.5 and 39.3 min, respectively. The equation (Q = cvt/V [31]) was utilized for the calculation of the loading capacity. In this equation, Q is the loading capacity (μg/cm^3^), c is the concentration of the analyte (mg/mL), v is the flow rate (mL/min), t is the saturation time (min), and V is the volume of monolithic column (cm^3^). When the flow rate was 10 μL/min, the loading capacity of 2,4-D on the above two monolithic columns were 499.3 and 801.0 μg/cm^3^, which illustrated the higher loading capacity of the obtained poly (OMA-co-TRIM) monolith. As a result, to gain a similar extraction efficiency of the analytes, the length of the poly (OMA-co-TRIM) monolith must be shorter than the poly (OMA-co-EDMA) monolith.

#### 2.1.4. Renewability

Apart from the residual CAHs, there are still many unknown things from the sample matrices of rice grains absorbed on the SPME monolith. After extraction, careful cleanup is necessary to make the SPME monolithic column renewable. In this work, ACN was utilized as the cleanup solution. The renewability of the SPME monolith was investigated by the decrease of the peak area of 2,4-D after several operation cycles. As represented in Figure S2, the decrease of the peak area on the poly (OMA-co-EDMA) monolith is bigger than the poly (OMA-co-TRIM) monolith, which denoted the better renewability of the poly (OMA-co-TRIM) monolith. This phenomenon should be attributed to the higher permeability and the higher loading capacity of the poly (OMA-co-TRIM) monolith. Herein, the length of the poly (OMA-co-TRIM) monolith used was shorter than the poly (OMA-co-EDMA) monolith. Due to the higher permeability and the shorter length of the poly (OMA-co-TRIM) monolith, a faster pump flow rate could be adopted for in-tube SPME. In this case, the contact time between the sample solution and the monolithic sorbent was shortened and led to the decrease of the absorbed impurities. On the other hand, owing to the higher hydrophobicity of the poly (OMA-co-TRIM) monolith, its extraction abilities toward CAHs was enhanced and further ensured the extraction efficiency. Therefore, the poly (OMA-co-TRIM) monolith should be a suitable sorbent for in-tube SPME of CAHs from rice grains.

### 2.2. Optimization of Some Important Parameters for Hydrophobic in-Tube SPME

#### 2.2.1. Length of the Monolithic Column

**Figure 3:**
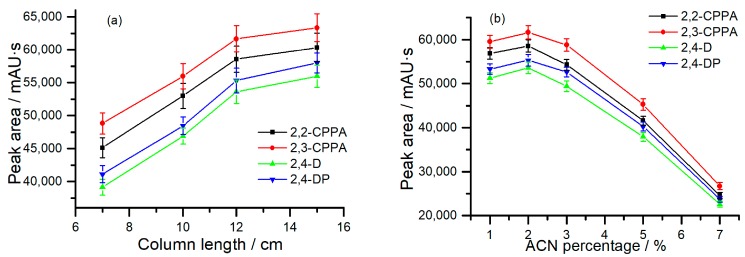
(**a**) Effect of the length of the monolithic column on the extraction efficiencies of the analytes; (**b**) effect of the ACN percentage in the sampling solution on the extraction efficiencies of the analytes. Abbreviation: 2-(2-chloro)-phenoxy propionic acid (2,2-CPPA), 2-(3-chloro)-phenoxy propionic acid (2,3-CPPA) and 2-(2,4-dichlorophenoxy)-propionic acid (2,4-DP).

The amount of monolithic sorbent depended on the length of the monolithic column. In this work, the length of the monolithic column was investigated from 7 to 15 cm. In Figure 3a, while keeping other parameters constant, the extraction efficiencies of the analytes increased with the length of the monolithic column. When the length increased up to 15 cm, the extraction efficiencies only increased a little. The results indicated that the sufficient extraction of CAHs could be obtained with 12 cm of the poly (OMA-co-TRIM) monolithic column. In addition, since the maximum of the pressure was constant in the system, using a short monolithic column meant that a faster flow rate was operated for the extraction, which led to less absorbed impurities, easier cleanup, and a shorter analytical time for the whole process. Thus, the length of 12 cm was chosen for the monolithic column in the following experiments.

#### 2.2.2. ACN Percentage in Sampling Solution

The ACN percentage in the sampling solution was a vital factor to affect the extraction behaviors of the hydrophobic compounds on the hydrophobic sorbent. The effect of the ACN percentage on the extraction efficiencies was investigated in the range of 1–7% (*v*/*v*). As seen in Figure 3b, the extraction efficiencies of the CAHs increased first and then decreased along with the increase in ACN percentage. The adsorption amount of CAHs (*n*) can be calculated by the equation *n* = K Vm c_0_ [32], in which K is the distribution coefficient, c_0_ is the initial concentration of the analytes, and Vm represents the volume of the monolithic stationary phase. Based on this equation, K and c_0_ influence the adsorption amount of CAHs. K decreases with the increase of the ACN percentage. When the ACN content was too low, the solubility of the CAHs was reduced, which caused the decrease of c_0_ and affected the final amount of the analytes. These two factors led to the result illustrated in Figure 3b. Therefore, the ACN percentage in the sampling solution of 2% was used for higher extraction efficiency.

#### 2.2.3. Trifluoroacetic Acid Percentage in Sampling Solution

**Figure 4:**
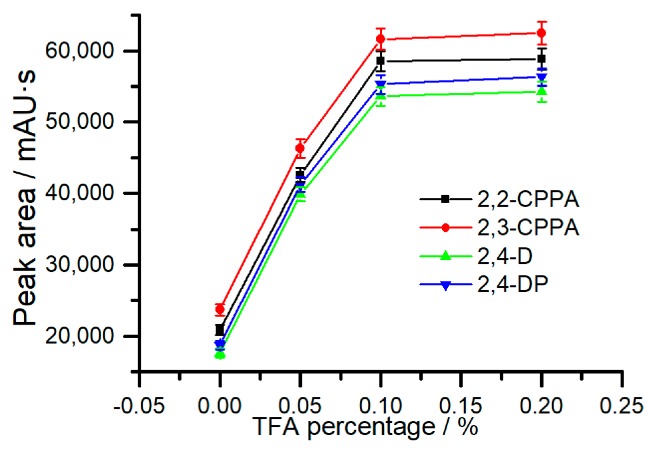
Effect of the trifluoroacetic acid (TFA) percentage in the sampling solution on the extraction efficiencies of the analytes.

Since the ionization state of CAHs can be controlled by the pH value of sampling solution, the percentage of trifluoroacetic acid (TFA) in the sampling solution is one of the most critical factors for their extraction. As shown in Figure 4, the extraction efficiencies of target compounds were significantly improved with the increase of TFA percentage from 0% to 0.1% (pH 2.02). When the TFA percentage was increased to 0.2% (pH 1.39), the extraction efficiencies were almost unchanged. With the increase of the TFA percentage in the sampling solution, the ionization degree of the acidic CAHs were reduced, leading to the enhancement of the hydrophobicities of the analytes and resulting in the increase of hydrophobic extraction efficiencies. Considering that the sampling solution with a high TFA percentage is harmful to the HPLC pump, the TFA percentage in the sampling solution was selected as 0.1% in subsequent experiments.

#### 2.2.4. Elution Volume

**Figure 5:**
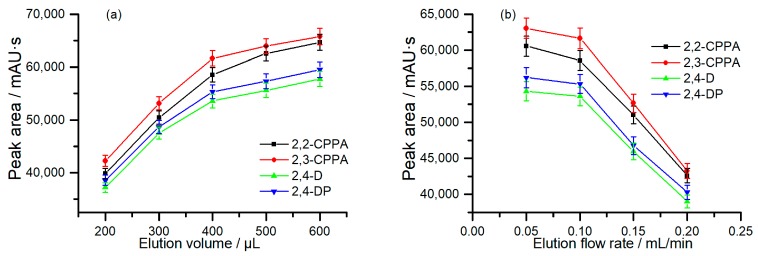
(**a**) Effect of elution volume on the extraction efficiencies of the analytes; (**b**) effect of elution flow rate on the extraction efficiencies of the analytes.

The elution volume is an important parameter affecting extraction efficiency due to its effect on the amount of the analytes injected into the HPLC column. Required by the online coupling system, the mobile phase (ACN: 0.1% TFA solution = 45:55, *v*/*v*) was directly applied as the elution solution for CAHs. The elution volume investigated ranged from 200 to 600 μL. As shown in Figure 5a, the extraction efficiency increased rapidly, with the elution volume increasing from 200 to 400 μL. Along with the increase in the elution volume, the extraction efficiency increased slowly. As a result, 400 μL was adopted as the optimal elution volume in this work.

#### 2.2.5. Elution Flow Rate

In the present work, the elution flow rate was investigated in the range of 0.05–0.2 mL/min. As can be seen in Figure 5b, the extraction efficiencies became worse with the increasing of the elution flow rate. This result might be attributed to that the faster elution flow rate can shorten the contact time between the poly (OMA-co-TRIM) monolithic column and the elution solution, leading to the decline of extraction efficiencies. Considering that the slow elution flow rate can extend the SPME time, as well as the total analysis time, 0.1 mL/min was utilized as the elution flow rate in the online hydrophobic in-tube SPME process.

### 2.3. Method Validation

**Figure 6:**
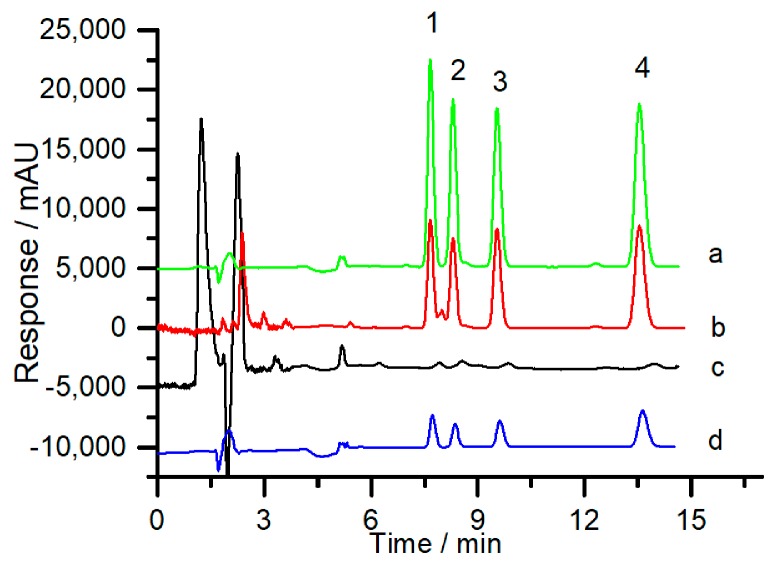
Chromatograms of four chlorophenoxy acid herbicides (CAHs) obtained from a spiked sample of rice grains (spiked at 200 μg/kg) by the proposed online hydrophobic in-tube solid-phase microextraction (SPME)-HPLC method (a) using the poly (OMA-co-TRIM) monolith as the sorbent, (b) using the poly (OMA-co-EDMA) monolith as the sorbent, (c) by direct injection mode of HPLC, and (d) obtained from practical sample of rice grains by the proposed online hydrophobic in-tube SPME-HPLC method using the poly (OMA-co-TRIM) monolith as the sorbent. SPME operating parameters: The length of the monolithic column, 12 cm; sampling solution, 0.1% TFA aqueous solution/ACN = 98/2 (*v*/*v*); sampling flow rate, 0.10 mL/min; sampling time, 5 min; elution flow rate, 0.10 mL/min; elution volume: 400 μL. Peak identifications: 1: 2,2-CPPA; 2: 2,3-CPPA; 3: 2,4-D; 4: 2,4-DP.

Under the optimized conditions, the analytical performance of the developed online hydrophobic in-tube SPME method using the poly (OMA-co-TRIM) monolithic column as the sorbent was further evaluated. As shown in Figure 6a, the spiked CAHs in a blank sample of rice grains could be well extracted, separated, and detected. In comparison with the direct injection mode of HPLC (Figure 6c), a drastic peak height enhancement of target compounds and obvious reduction of matrix interference was achieved, which indicated the strong hydrophobic extraction effect and purification effect of the poly (OMA-co-TRIM) monolithic column. Herein, the enrichment factor (F_E_), which is defined as the ratio of the peak area of analytes with in-tube SPME-HPLC to that with the direct injection mode of HPLC, was utilized to evaluate the extraction effect of the poly (OMA-co-TRIM) monolith. As listed in Table 1, the F_E_ values of the analytes ranged from 83.6 to 98.3, which indicated the excellent hydrophobic extraction effect of the poly (OMA-co-TRIM) monolith. By using the poly (OMA-co-EDMA) monolithic column as the SPME sorbent (Figure 6b), a lower peak height of the analytes and distinct baseline noise from sample matrix occurred. It is worth noting that, in this case, the first two analytes could not be well separated due to the existence of something unknown from the extracted elution. These results confirmed the unsatisfied extraction efficiency and the inapplicability of poly (OMA-co-EDMA) monolith facing the complex samples of rice grains.

The linearity, linear range, limits of detection (LODs), and correlation coefficients (R^2^) were determined for four CAHs in the samples of rice grains and listed in Table 1. The linear range was in the range of 5–600 μg/kg for four CAHs with correlation coefficients ranging from 0.9957 to 0.9996. LODs were detected as the concentration of the analytes at S/N = 3 and ranged from 0.9 to 2.1 μg/kg, which could meet the requirement of the standards [13,33,34] when determining trace amount of CAHs. The recovery studies were carried out with blank samples of rice grains being spiked with the CAHs at three concentrations of 25, 150, and 250 μg/kg, respectively. For each concentration level, three replicate experiments were made. As summarized in Table 2, the recoveries were in the range of 87.3–111.6%, with relative standard deviations (RSDs) less than 7.3%. These results demonstrated the strong applicability of the poly (OMA-co-TRIM) monolith, as well as the excellent sensitivities and recoveries of the proposed method for analysis of CAHs residue in rice grains.

### 2.4. Reproducibility

The reproducibility of the poly (OMA-co-TRIM) monolith for hydrophobic in-tube SPME was investigated with respect to the peak areas of four CAHs. As listed in Table 3, while using the same one monolithic column for five replicate extractions, the resultant RSDs ranged from 0.6% to 2.5%. The batch-to-batch reproducibility for the poly (OMA-co-TRIM) monoliths was also evaluated in terms of the RSDs of the peak areas of the analytes, with the RSDs being less than 9.3%. The above results indicated that good reproducibility and repeatable extraction performance of the developed monolithic columns.

### 2.5. Comparison of the Proposed Method with the Standard Method of LC-MS

The application of the proposed method was further evaluated by the comparison with the standard method of LC-MS [14]. As listed in Table 4, the LODs of the proposed method were closed to the standard method of LC-MS, while the analytical time was shortened to a third of the standard method, indicating the briefness and sensitivity of the online hydrophobic in-tube SPME-HPLC method using poly (OMA-co-TRIM) monolithic column as the sorbent.

The proposed method was applied to determine the trace CAHs (2,2-CPPA, 2,3-CPPA, 2,4-D and 2,4-DP) in practice rice grains (Figure 6d). Rice grain samples, which were sprayed with these herbicides during planting, were quantified and calculated. The residual contents of 2,2-CPPA, 2,3-CPPA, 2,4-D, and 2,4-DP detected by the proposed online hydrophobic in-tube SPME-HPLC method were 19.8 μg/kg, 21.4 μg/kg, 29.3 μg/kg, and 34.6 μg/kg, respectively, while the values detected by the standard method of LC-MS were 22.3 μg/kg, 20.1 μg/kg, 29.4 μg/kg, and 38.4 μg/kg, respectively. This shows the strong reliability of the proposed method. Furthermore, all the residual contents of the CAHs were lower than the maximum residue limits (MRLs) in the standards of different countries. This means that the CAHs residues meet the standard requirements, while the CAHs were sprayed according to the instructions.

## 3. Materials and Methods

### 3.1. Chemicals and Materials

2,4-Dichlorophenoxyacetic acid (2,4-D), 2-(2,4-dichlorophenoxy)-propionic acid (2,4-DP), 2-(2-chloro)-phenoxy propionic acid (2,2-CPPA), and 2-(3-chloro)-phenoxy propionic acid (2,3-CPPA) were all purchased from Sigma (St. Louis, MO, USA). OMA, TRIM, 2,2-azobisisobutyronitrile (AIBN), and 3-(trimethoxysilyl)-propyl methacrylate (γ-MAPS) were bought from Acros (Morris Plains, NJ, USA). Cyclohexanol and 1,4-butanediol were supplied by Tianjin Guangfu Fine Chemical Research Institute (Tianjin, China). Deionized water was obtained by using a Millipore Milli-Q purification system (Milford, MA, USA). Acetonitrile (ACN) and methanol (Chemical Reagent Corporation, Shanghai, China) were of HPLC grade. Trifluoroacetic acid (TFA), hydrochloric acid, sodium hydroxide, ammonium acetate, magnesium sulphate, and ethylene diamine-*N*-propyl silane (PSA) were purchased from Shanghai Chemical Reagents Corporation (Shanghai, China). Herein, 0.1 mol/L hydrochloric acid solution and 0.1 mol/L sodium hydroxide solution were used for the pre-treatment of the capillary inner wall, according to the literature [35]. The fused-silica capillaries with dimensions of 250 μm i.d. were obtained from Refine Chromatography Ltd. (Yongnian, China). The rice seeds were afforded by the College of Crop Science, Fujian Agriculture, and Forestry University (Fuzhou, China).

### 3.2. Apparatus

The HPLC-DAD system (Shimadzu, Kyoto, Japan), consisting of a Shimadzu LC-20AD pump, a Shimadzu SPD-M20A photodiode array detector, and a Shimadzu DGU-20A5 degasser, was employed for determining CAHs. Shimadzu LCsolution software was used for system control and data analysis. The HPLC conditions for this work were referred to our previous work [29]. ACN/0.1% TFA solution (45:55, *v*/*v*) was introduced as the mobile phase, eluting the analytes at 1 mL/min for HPLC analysis. The detection wavelength was set at 223 nm and the temperature of in-tube SPME and HPLC was set at 30 °C. A Gemini 5u C18 column (250 × 4.6 mm) from Phenomenex Inc. (Torrance, CA, USA) was used for the separation. 

The LC-MS analysis was performed with an Agilent 1200/6460 LC-MS system (Palo Alto, CA, USA) with electron spray ionization in the negative ionization mode. A 150 × 2.1 mm Gemini 5u C18 column was used for separation with a flow rate of 0.3 mL/min and column temperature of 40 °C. A 20 μL injection volume was used. A solvent gradient between A = 5 mmol/L ammonium acetate methanol solution and B = 5 mmol/L ammonium acetate aqueous solution was used. The gradient elution program was as follows: From 0 to 30 min, A was increasing from 10% to 80%, while B was decreasing from 90% to 20%. The calculated molecular ions (198, 198, 219, 233 mz^−1^) of the analytes were collected.

An LC-10AD pump (Shimadzu, Kyoto, Japan) was utilized to construct the online hydrophobic in-tube SPME-HPLC system and rinse the monolithic column. GL-16 centrifuge with highest speed of 16,000 rpm was purchased from Anting Technology (Shanghai, China).

### 3.3. Preparation of the Poly (OMA-co-TRIM) Monolithic Column

In order to improve the stability of the monolithic columns, the inner wall of a capillary was treated with a bifunctional reagent, γ-MAPS, according to the procedure reported previously [35]. The pre-polymerization mixture consisted of a monomer OMA (360 mg, 18%, *w*/*w*), a crosslinker TRIM (240 mg, 12%, *w*/*w*), porogenic solvents cyclohexan (1120 mg, 56%, *w*/*w*), 1,4-butanediol (280 mg, 14%, *w*/*w*), and initiator AIBN (1.8 mg, 0.3%, *w*/*w*, with respect to monomer and crosslinker). After purging with a N_2_ stream for 30 min to remove the oxygen, the mixture was allowed to fill in the capillary. The capillary was immediately sealed at both ends with rubbers and the reaction was initiated in a water bath at 60 °C for 24 h. The prepared monolithic capillary was washed with methanol to remove the unreacted components and porogenic solvents.

### 3.4. Rice Cultivation

Breeding started in late April. In late May, the seedlings were transplanted to the two isolated paddy fields (one for CAHs-sprayed and one for control). According to the farmers' normal tillage requirements, the fertilizers used were urea, phosphate, potash, and ammonium hydrogen carbonate. The CAHs were sprayed between the tillering stage and the jointing stage, with a dosage of 1.0 kg/ha (about twice the normal dosage). The rice grains were collected after rice maturity.

### 3.5. Sample Preparation

The samples of the rice grains were prepared based on the method from the agriculture standards of the People’s Republic of China (NY/T 1434-2007) [14], with some modifications. Approximately 50 g of rice grains were ground at room temperature. A portion of 15 g ground sample was weighed into a 50 mL Teflon centrifuge tube and covered by 30 mL of ACN-TFA (99.9:0.1, *v*/*v*) and 6 g MgSO_4_. Next, the mixture was shaken by the vortex mixer for 1 min. The sample extract was centrifuged at 4000 rpm for 1 min. 10 mL of upper layer extract was then transferred to a 15 mL centrifuge tube containing 0.6 g MgSO_4_ and 0.25 g PSA. The mixture was vortexed for 1 min and centrifuged at 7000 rpm for 1 min. After centrifugation, 3 mL of the supernatant was collected and dried under N_2_ stream at 50 °C. Finally, the dried residues were reconstituted with a solution containing 0.1% TFA solution (98%, *v*/*v*) and ACN (2%, *v*/*v*) (the solution of MS was ACN) to bring the final sample volume to 3 mL. Finally, the sample solution was filtered by using 0.22 μm needle filters for analysis.

### 3.6. Online Hydrophobic in-Tube SPME-HPLC System

The online hydrophobic in-tube SPME-HPLC system was constructed on the basis of our previous works [36]. The poly (OMA-co-TRIM) monolithic column (12 cm × 0.25 mm, i.d.) was used as microextraction sorbent, the schematic diagram of the online hydrophobic in-tube SPME-HPLC system used for the study was illustrated in Figure S3 and the procedure used for this online system is described in the supplementary material.

## 4. Conclusions

In this work, a novel poly (OMA-co-TRIM) monolithic column was specially prepared and utilized as a hydrophobic sorbent for online hydrophobic in-tube SPME. Due to the excellent extraction and purification effects towards hydrophobic analytes, the developed monolithic column showed its applicability for hydrophobic microextraction. After the optimization of the important factors for in-tube SPME, a simple and sensitive online in-tube SPME-HPLC method using this poly (OMA-co-TRIM) monolithic column as sorbent was proposed for the determination of trace CAHs in rice grains. Low detection limits (0.9–2.1 ng/g) for four CAHs were obtained and the sensitivity of the proposed method could reach that of LC-MS. Moreover, this work shows that hydrophobic in-tube SPME based on this poly (OMA-co-TRIM) monolith could be a feasible approach for the determination of hydrophobic target compounds in complex practical samples.

## Figures and Tables

**Table 1:** Linearity, linear range, R^2^, LOD and F_E_ of analytes in the samples of rice grains.

Analytes	Linearity ^1^	R^2^	Linear Range (μg/kg)	LOD (μg/kg)	F_E_
2,2-CPPA	Y = 533.0x + 1060.7	0.9957	5–600	1.5	85.7
2,3-CPPA	Y = 507.4x + 1017.7	0.9977	5–600	2.1	83.6
2,4-D	Y = 554.6x + 513.4	0.9973	5–600	1.2	96.7
2,4-DP	Y = 592.2x + 563.4	0.9996	5–600	0.9	98.3

^1^ x, Concentration of analyte (μg/kg); y, peak area (mAU·s).

**Table 2:** Recoveries of four CAHs in rice grains.

Analyte	Spiked/(μg/kg)	Found/(μg/kg)	Recovery ^1^/%	RSD/% (*n* = 3)
2,2-CPPA	25	21.8	87.3	5.2
	150	142.3	94.9	2.7
	250	254.0	101.6	1.2
2,3-CPPA	25	24.6	98.4	4.7
	150	134.0	89.3	2.3
	250	259.0	103.6	0.6
2,4-D	25	27.9	111.6	6.9
	150	151.2	100.8	3.7
	250	232.3	92.9	1.8
2,4-DP	25	26.4	105.6	7.3
	150	145.4	96.9	5.5
	250	244.3	97.7	3.4

^1^ Recovery = found/spiked × 100%.

**Table 3:** RSDs for the peak areas on different poly (OMA-co-TRIM) monoliths.

Analytes	Run-to-Run Reproducibility (*n* = 5)	Batch-to-Batch Reproducibility (*n* = 3)
2,2-CPPA	1.3	7.9
2,3-CPPA	0.6	6.9
2,4-D	1.9	5.1
2,4-DP	2.5	9.3

**Table 4:** Comparison of the proposed online hydrophobic in-tube SPME-HPLC method with the standard method of LC-MS.

Methods	Retention Time (min)				LOD (μg/kg) ^1^			
	2,2-CPPA	2,3-CPPA	2,4-D	2,4-DP	2,2-CPPA	2,3-CPPA	2,4-D	2,4-DP
This method	8.2	8.7	10.1	14.3	1.2	0.9	2.1	1.5
LC-MS	24.8	26.1	26.2	27.0	-	-	3.0	1.0

^1^ The LOD values of HPLC-MS were found from the agriculture standards of the People’s Republic of China (NY/T 1434-2007).

## Supplementary Materials

The following are available online: Figure S1: Pore size distribution profile of the poly (OMA-co-TRIM) monolith by the mercury intrusion method; Figure S2: The peak area of 2,4-D after several operation cycles; Figure S3: Construction of the online hydrophobic in-tube SPME-HPLC system.
