# Supplementary material for: Poly (Octadecyl Methacrylate-Co-Trimethylolpropane Trimethacrylate) Monolithic Column for Hydrophobic in-Tube Solid-Phase Microextraction of Chlorophenoxy Acid Herbicides
Source: Molecules. 2019 Apr 29;24(9):1678. doi: 10.3390/molecules24091678 (PMC6540311; doi:10.3390/molecules24091678)
Supplement: Supplementary file 1 [file molecules-24-01678-s001.pdf]

molecules

Supplementary Materials

Poly (Octadecyl Methacrylate-Co-Trimethylolpropane Trimethacrylate) Monolithic Column for Hydrophobic in-Tube Solid-Phase Microextraction of Chlorophenoxy Acid Herbicides

Wenbang Li [1], Fangling Wu [1], Yongwei Dai [2], Jing Zhang [3], Bichen Ni [1] and Jiabin Wang [1,*]

[1] Institute of Biomedical and Pharmaceutical Technology, Fuzhou University, Fuzhou 350002, China; N165720012@fzu.edu.cn (W.L.), flwu16@fudan.edu.cn (F.W.); N185720006@fzu.edu.cn (B.N.)
[2] Fuzhou Jiachen Biotechnology Co., Ltd., Fuzhou 350008, China; daiyoungwei@163.com
[3] Fujian Inspection and Testing Centre for Agricultural Product Quality and Safety, Fuzhou 350003, China; fjsy0591@sina.com
* Correspondence: jbwang@fzu.edu.cn; Tel.: +86-591-8372-5260

1. Porosity

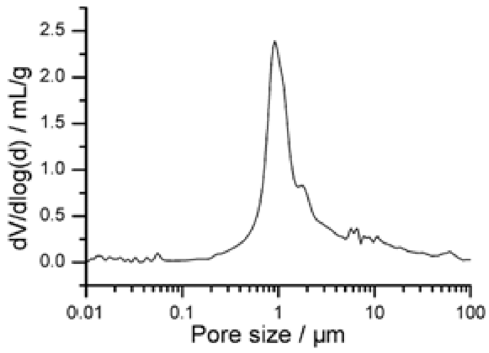

Figure 1. Pore size distribution profile of the poly (OMA-co-TRIM) monolith by the mercury intrusion method.

2. Renewability

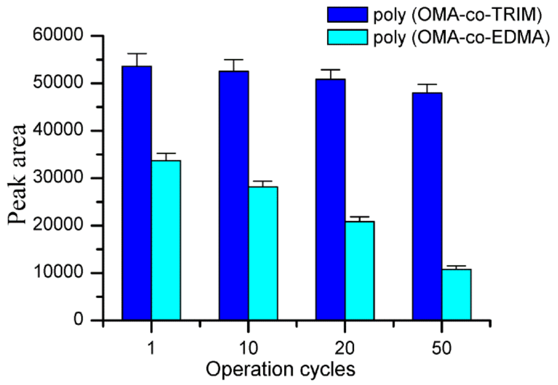

Figure 2. The peak area of 2,4-D after several operation cycles.

3. Construction of the online hydrophobic in-tube SPME-HPLC system

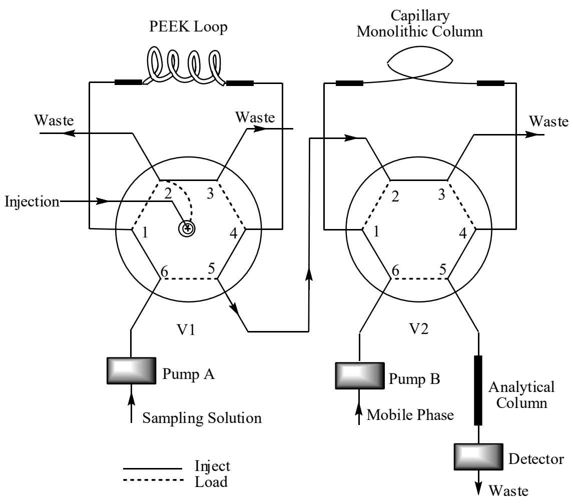

Figure 3. Construction of the online hydrophobic in-tube SPME-HPLC system.

A 0.5 mL PEEK loop was mounted on valve 1. The prepared hydrophobic poly (OMA-co-TRIM) monolithic column was installed on valve 2 in the position where the loop was originally positioned.

Before extraction, valves 1 & 2 were initially set at LOAD positions. The sampling solution (0.1% TFA aqueous solution/ACN = 98/2 (v/v)) was driven by pump A to flow through the monolithic column for conditioning at 0.10 mL/min. The mobile phase was driven by pump B directly through the analytical column to obtain a stable baseline for chromatographic separation. Meanwhile, the PEEK loop was filled with the sample solution using a syringe.

When extraction began, valve 1 was directed towards INJECT position for a given time (5 min) and returned to LOAD position immediately to perform extraction. The sampling solution was kept to flow through the monolithic column for 90 s in order to eliminate the residual sample solution and reduce the interference.

Then, the extracted analytes were eluted from the monolithic column by the mobile phase at a flow rate of 0.10 mL/min by simply switching the valve 2 to the INJECT position. When extraction had finished, valve 2 was switched to the LOAD position, and followed by adjusting the flow rate of the mobile phase to 1.0 mL/min for separation. Meanwhile, the monolithic column for SPME was rinsed with ACN for 20 min by pump A at a flow rate of 0.10 mL/min to make the SPME monolithic column renewable.

4. Recovery studies

In this work, the analytes were spiked into the blank samples of rice grains for recovery studies. After spiked by different amounts of the analytes, these samples of rice grains were prepared as Section 3.5 to obtain the sample solutions for analysis. The found concentrations of the analytes were calculated by the working curves in Table 1. Thus, the recoveries of the analytes was calculated by using the equation (viz. Recovery = Found concentration / Spiked concentration ×100%).
